# The Use of Recycled PET for the Synthesis of New Mechanically Improved PVP Composite Nanofibers

**DOI:** 10.3390/polym14142882

**Published:** 2022-07-16

**Authors:** Manuel A. Gallardo-Sánchez, Manuel J. Chinchillas-Chinchillas, Alberto Gaxiola, Clemente G. Alvarado-Beltrán, Abel Hurtado-Macías, Víctor M. Orozco-Carmona, Jorge L. Almaral-Sánchez, Selene Sepúlveda-Guzmán, Andrés Castro-Beltrán

**Affiliations:** 1 Universidad de Guadalajara, Centro Universitario de Ciencias Exactas e Ingenierías, Guadalajara C.P. 44430, Jalisco, Mexico; manuel.gallardo@academicos.udg.mx; 2 Universidad Autónoma de Occidente (UAdeO), Unidad Regional Guasave, Departamento de Ingeniería y Tecnología, Los Mochis 81048, Sinaloa, Mexico; manuel.chinchillas@uadeo.mx; 3 Universidad Autónoma de Sinaloa, Facultad de Ingeniería Mochis, Los Mochis C.P. 81223, Sinaloa, Mexico; alberto.gaxiola@uas.edu.mx (A.G.); calvarado@uas.edu.mx (C.G.A.-B.); jalmaral@uas.edu.mx (J.L.A.-S.); 4 Centro de Investigación en Materiales Avanzados, Chihuahua C.P. 31136, Chihuahua, Mexico; abel.hurtado@cimav.edu.mx; 5 Universidad Autónoma de Nuevo León, Facultad de Ingeniería Mecánica y Eléctrica, San Nicolás de los Garza C.P. 66451, Nuevo León, Mexico; selene.sepulvedagz@uanl.edu.mx

**Keywords:** recycled PET, polyvinylpyrrolidone, composite nanofibers, glycolysis, crosslinking, nanoindentation

## Abstract

Polyethylene terephthalate (PET) waste has become a major challenge for the conservation of the environment due to difficult degradation. For this reason, it is important to develop new recycling strategies for reusing this waste. In this work, the electrospinning technique was used to synthesize composite nanofibers of polyvinylpyrrolidone (PVP), recycling PET (RPET) that was obtained from the chemical recycling of postconsumer PET with glycolysis and styrene (ST) as a crosslinking agent. The polymer solutions were analyzed by viscosity and frequency sweeping, while the composite nanofibers were characterized by scanning electron microscopy (SEM), Fourier transform infrared spectroscopy (FTIR), thermogravimetric analysis/differential scanning calorimetry (TGA/DSC), and nanoindentation to compare their properties. The PVP nanofibers presented an average diameter of 257 nm; the RPET/PVP and RPET/PVP/ST composite nanofibers had average diameters of 361 nm and 394 nm, respectively; and the modulus of elasticity and hardness of the RPET/PVP/ST composite nanofibers were 29 and 20 times larger, respectively, than those of the PVP nanofibers. With the synthesis of these composite nanofibers, a new approach to PET recycling is presented.

## 1. Introduction

The growing production of polyethylene terephthalate (PET) bottles has become a major problem in recent years. In 2014, the world consumption of PET bottles was approximately 42 Mt, while for 2020, it was estimated to be approximately 73 Mt, with an annual increase of 6.2% [1,2,3]. Despite the wide use of PET, a very small amount has been recycled due to the low value of the material and the economic infeasibility of its recycling [4]. Certainly, there are many benefits of recycling these materials, such as reductions in greenhouse gases, water/air pollution, energy consumption, and conservation of natural resources [5,6]. Mechanical and chemical methods are the two main ways to recycle PET bottle waste. In mechanical methods, PET bottle waste is crushed into very fine scraps, which are subsequently heated, melted, and remodeled into new products [7]. The chemical recycling of PET bottle waste consists of depolymerization to obtain the initial monomers by hydrolysis, methanolysis, aminolysis, or glycolysis [8,9]. Glycolysis has been established to be the most cost-effective method for chemical recycling of PET bottle waste because it requires low amounts of reactants, as well as lower temperatures and pressures than other methods [10,11]. The obtained products are monomers and oligomers that can successfully be used as precursors in the production of recycled PET (RPET) [12]. The reinforcement of materials such as cementitious matrices [13], coatings [14], biomedicine, and tissue engineering [15] are some examples of applications of RPET. However, its applications are very limited due to the low value of its mechanical properties. For this reason, it is of vital importance to improve its mechanical performance to increase the range of possible applications. One way to improve the mechanical properties is by crosslinking the RPET molecules through an agent such as ST [16]. ST has been used as a polymer crosslinking agent because it is cheap and improves polymer properties such as stiffness, mechanical strength, and thermal stability [17]. Another way to improve the mechanical properties of RPET is to chemically bond RPET and a virgin polymer to produce a composite material [18].

PVP is an important amorphous polymer with low chemical toxicity, high biocompatibility, excellent solubility in most organic solvents, good spinnability, and the capability to interact with a wide range of hydrophilic materials [19,20]. Due to its good properties, PVP is widely used to prepare composite materials since it can be chemically linked to other polymers using a reaction initiator (potassium persulfate) and a catalyst (temperature) [21].

Composite nanofibers are versatile and have been studied because of their interesting potential applications. They are known for their high surface/volume ratios and higher mechanical performance compared with macroscale fibers; this aspect represents a potential value-added application for waste plastics [22]. Nanofibers are used in a wide variety of research and commercial areas, including filtration, reinforcement of cementitious matrices, tissue culture, medical materials, and as filler in composite materials [23,24,25]. Among the various techniques that have been developed for the production of nanofibers, electrospinning has attracted attention in recent decades because it is a simple, convenient, and versatile method for the manufacture of fibers with good morphology and diameters that range from ~10 nm to ~1 µm [26]. In the literature, it has been reported that composite PVP nanofibers with other materials, such as polyacrylonitrile [27], the luminescent europium complex [28], ZnO [29], poly(vinylidene fluoride) [30], silica [31], Ag nanoparticles [32], borneol [33], and TiO_2_ [34], show improved thermal and mechanical properties compared with PVP. However, PVP has not been used to form composite nanofibers with RPET. According to the literature, the only polymer that has been used to form composite nanofibers with RPET is polyacrylonitrile (PAN) [35].

In this research, the electrospinning technique was used to synthesize composite nanofibers of PVP, RPET (which was obtained from chemical recycling of postconsumer PET bottles by glycolysis), and ST. The composite nanofibers of RPET/PVP/ST were characterized by SEM, FTIR, TGA/DSC, and nanoindentation to analyze their morphology, structure, thermal behavior, and mechanical properties.

## 2. Materials and Methods

### 2.1. Materials

The following materials were used in this investigation: recycled PET bottles (obtained from various recycling companies in Mexico), sodium hydroxide (99% purity, Fraga Lab., Los Mochis, Mexico), propylene glycol (99.99% purity, Sigma–Aldrich, Los Mochis, Mexico), zinc acetate (99.99% purity, Sigma–Aldrich, Los Mochis, Mexico), ST (99% purity, Sigma–Aldrich, Los Mochis, Mexico), maleic anhydride (MA) (99% purity, Sigma–Aldrich, Los Mochis, Mexico), adipic acid (AA) (99% purity, Sigma–Aldrich, Los Mochis, Mexico), PVP (Mw = 1300.000 g·mol^−1^, Sigma–Aldrich, Los Mochis, Mexico), potassium persulfate (KPS, 99.0% purity), and dimethylformamide (DMF, 99.85%, CTR Scientific, Los Mochis, Mexico).

### 2.2. Preparation of the Nanofibers

RPET was synthesized using the procedure that was reported by Mendivil (2017) [11]. First, postconsumer PET bottles were crushed using a nonstandard scissor cutting blade mill with a power of 5 hp. After the grinding treatment, the obtained PET flakes, which had a diameter of approximately 1.3 mm, were washed in caustic soda to remove impurities. The PET flakes were dried at room temperature and added to a vessel reactor with 50 wt.% propylene glycol and zinc acetate at 0.5 wt.%. The temperature was increased from 25 °C to 197 °C at a rate of 4 °C/min, and the mixture was kept under agitation (180 rpm) for 3 h. The resulting material was labeled BHET. Finally, BHET, AM, and AA in a molar ratio of 1.1:0.5:0.5 were mixed in the same reactor under the same conditions to obtain RPET.

A PVP solution (20 wt.%) was prepared in DMF. A homogeneous solution of RPET/PVP in DMF was prepared by adding 20 wt.% PVP, 10 wt.% RPET, and 5 wt.% KPS with respect to the weight of RPET. A solution of RPET/PVP/ST in DMF was prepared by mixing 20 wt.% PVP, 10 wt.% RPET dissolved in 30% ST, and 5% KPS by weight of RPET.

**Figure 1:**
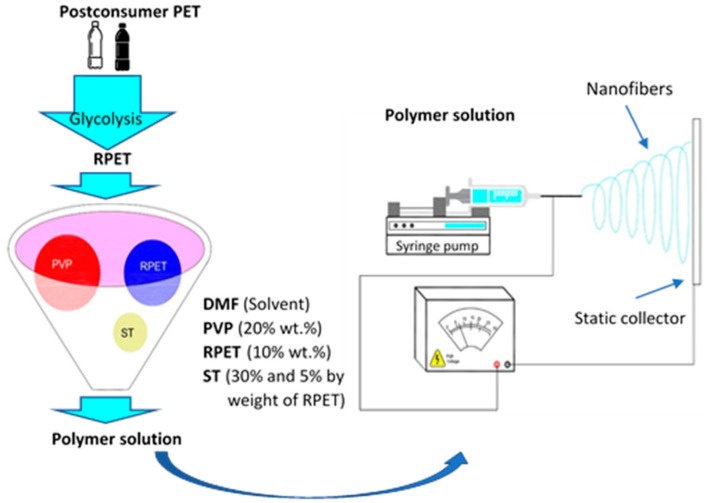
Schematic of the preparation of the RPET/PVP/ST nanofibers.

In the electrospinning process, a high-voltage source (Model ES30P-5W/DAM, Gamma High Voltage brand) was used. An infusion pump (Model NE-300, Syring Pumb brand) was utilized, and commercial aluminum sheets were placed in the collector of the equipment. The equipment was configured vertically, and the solution was placed in a 5 mL syringe with a capillary tip (needle) that was 1.25 mm in diameter. For the PVP, RPET/PVP, and RPET/PVP/ST solutions, the applied voltage was 20 kV, the distance between the tip and the manifold was 20 cm, and the flow rate of the spun solution was 0.5 mL/h. The nanofibers were collected on aluminum foil that was supported in a static collector. Finally, the RPET/PVP and RPET/PVP/ST nanofibers were thermally treated in a SHEL Lab Model CE5F oven in an oxygen environment at 80 °C for 24 h to cause crosslinking among the RPET, PVP, and ST. This process is shown in Figure 1.

### 2.3. Characterization

To determine the characteristics of the polymeric solution, a dynamic shear rheometer (Model MCR501 TruGap, Anton Paar) was used at Centro de Investigación en Materiales Avanzados (CIMAV, Chihuahua, Mexico). The viscosity was determined as a function of the shear rate (within the range of 0.001 to 100 s^−1^), and an oscillatory frequency sweep was conducted in the linear viscoelastic range at 25 °C, with a concentric cylinder geometry with a size of 27 mm. In this study, the polymeric solutions were subjected to the same thermal treatment as the nanofibers. The morphology of the nanofibers was observed by SEM using an FEI Nova NanoSEM microscope (CIMAV, Chihuahua, Mexico) with a working distance of 10 mm and a voltage of 5 kV. In the measurements, a low-vacuum detector was used, since the samples were not conductive. To obtain the average diameter, ImageJ software was used to determine the diameters of 110 nanofibers that were chosen randomly from the micrographs. The starting materials and the nanofibers were analyzed by FTIR spectroscopy using a Bruker-Alpha II instrument at Universidad Autónoma de Sinaloa (UAS, Los Mochis, Mexico); scanning was conducted from 4000 cm^−1^ to 500 cm^−1^ with a resolution of 4 cm^−1^. The thermal behavior (TGA/DSC) of the nanofibers was investigated using a thermal analyzer (SDT Q600, TA Instrument, UAS, Los Mochis, Mexico). The analysis was performed with a 5–10 mg sample in a dynamic nitrogen atmosphere with a heating rate of 10 °C/min from room temperature to 800 °C. To analyze the mechanical properties of the nanofibers, the nanoindentation technique was used to obtain the elastic modulus (E) and the hardness (H) using a Nanoindenter G200 instrument that was coupled to a DCM II head (CIMAV-México). It was necessary to calibrate the equipment with a standard sample of fused silica to begin the measurements. The tests were conducted in a 3 × 3 cm nanofibrous membrane matrix under the following experimental conditions: a Berkovich diamond indenter with a tip radius of 20 ± 5 nm, a maximum load of 2 mN, a strain rate of 0.05 s^−1^, a Poisson’s coefficient of ν = 0.25, a loading time of 10 s, and a Poisson ratio of 0.18.

## 3. Results and Discussion

### 3.1. Rheology of the Solution

**Figure 2:**
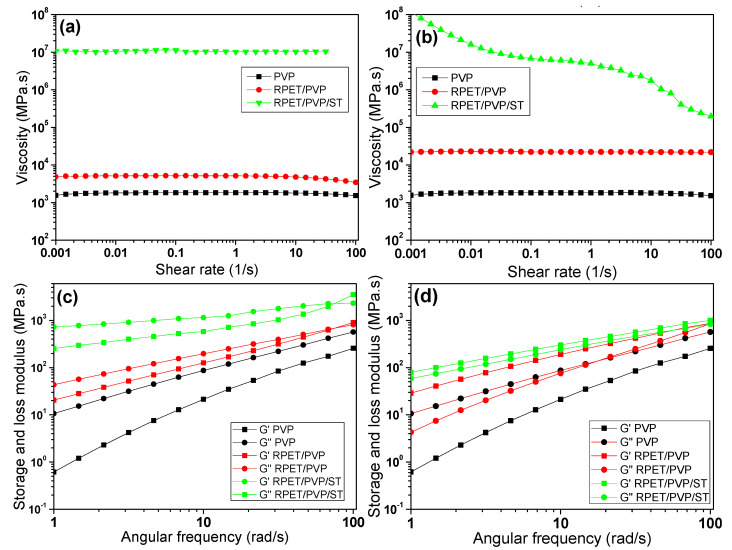
Rheology of the polymer solutions: (**a**,**b**) viscosity and (**c**,**d**) frequency sweep test results before and after thermal treatment, respectively.

The viscosity is a critical parameter for determining the morphology of electrospun fibers. Figure 2 shows the viscosity and frequency sweep test results of the PVP, RPET/PVP, and RPET/PVP/ST solutions before and after the thermal treatment. The three solutions showed Newtonian behavior, with a constant viscosity over the entire frequency range (Figure 2a). The PVP solution showed a viscosity of 1821.2 MPa·s, the RPET/PVP solution had a viscosity of 5199.7 MPa·s, and the RPET/PVP/ST solution had a viscosity of 10.927 MPa·s. The increases in the viscosity of the compound solutions with respect to PVP were due to the higher RPET concentration (10% wt.) in these solutions; however, in the case of the RPET/PVP/ST solution, the viscosity increased very significantly because the solution contained ST, which helped crosslink the polymer chains [36]. After the heat treatment, the PVP solution showed no change in viscosity. However, the viscosity of the RPET/PVP solution increased to approximately 2240 MPa·s. Additionally, it was observed that in the RPET/PVP/ST solution, the viscosity was not constant, which is characteristic of a non-Newtonian material (gel-solid), because the temperature acted as a catalyst that produced free radicals from the KPS, thereby promoting crosslinking among the RPET, PVP, and ST. This crosslinking caused the polymer chains to become stiffer and less resistant to shear. Figure 2c shows that the viscous component (G″) predominated over the elastic component (G′) in the PVP solution and that throughout the angular frequency range, the component curves did not cross; this behavior is typical of a viscous liquid [37]. The curves of the RPET/PVP and RPET/PVP/ST solutions crossed at ~2260 MPa·s, thereby indicating that they have a higher molecular weight than the PVP solution [38,39,40,41]. After the heat treatment (Figure 2d), the viscoelastic behavior of the PVP solution did not change, but for the RPET/PVP and RPET/PVP/ST solutions, G′ was above G″, which indicates that these solutions behaved more like solid materials than viscous materials. This was because the temperature accelerated the crosslinking among RPET/PVP/ST, thereby enabling the formation of a composite material [42,43].

### 3.2. SEM

**Figure 3:**
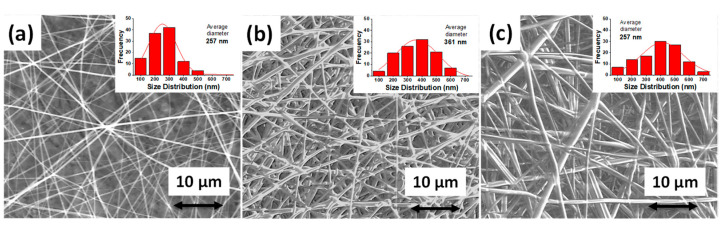
SEM images of (**a**) PVP nanofibers, (**b**) RPET/PVP nanofibers, and (**c**) RPET/PVP/ST nanofibers.

The morphologies of the PVP, RPET/PVP, and RPET/PVP/ST nanofibers that were obtained by electrospinning were observed using a scanning electron microscope and are shown in Figure 3. The PVP nanofibers were smooth, free of defects, and randomly oriented, with an average diameter of 257 nm and a standard deviation of 5.3 nm (Figure 3a). Another investigation reported a similar morphology and diameter with the same electrospinning parameters [29]. For the RPET/PVP sample, nonuniform nanofibers that were randomly distributed and interlaced with one another were observed, with an average diameter of 361 nm and a standard deviation of 10.03 nm (Figure 3b), which is approximately 100 nm greater that the diameter of the PVP nanofibers. The RPET/PVP/ST nanofibers were more uniform; some of them were linked together, with an average diameter of 394 nm and a standard deviation of 17.83 nm (Figure 3c). The composite nanofibers were larger because they contained RPET, which increased the viscosity (as shown in Figure 2a); it is well known that the higher the viscosity of a polymer solution, the larger the diameter of the nanofibers [44,45].

### 3.3. FTIR

**Figure 4:**
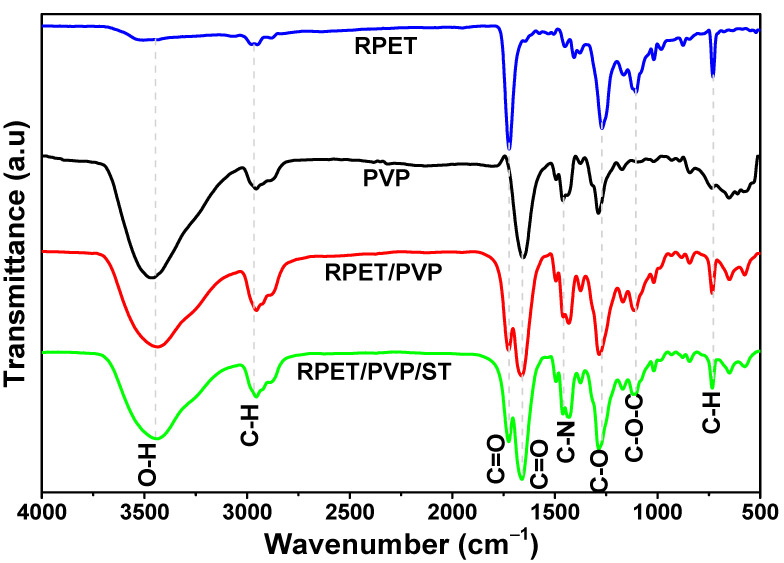
Infrared spectra of RPET, PVP, RPET/PVP, and RPET/PVP/ST nanofibers.

Figure 4 shows the FTIR spectra that were obtained for RPET and the PVP, RPET/PVP, and RPET/PVP/ST nanofibers. The RPET spectrum showed a band at approximately 3442 cm^−1^, which is characteristic of O-H groups, as well as a band at 2960 cm^−1^, which is attributed to the asymmetric stretching mode of the C-H bond. Additional bands were also localized at 1720 cm^−1^, 1280 cm^−1^, 1100 cm^−1^, and 730 cm^−1^, which correspond to the carbonyl group (C=O), stretching of the C-O band, the C-O-C bond, and the out-of-plane stretching of C-H, respectively. Notably, all the identified bands are characteristic of RPET [46]. In the FTIR spectrum of the PVP nanofibers, a band at 3440 cm^−1^ was observed, which is attributed to the OH groups that were present in the sample due to the moisture that was absorbed by PVP [47]. At 2940 cm^−1^, a band corresponding to the asymmetric tension vibration of C-H was observed, while the vibration of the C=O bond was observed at 1660 cm^−1^ and corresponds to the carbon bonded with the oxygen within the aromatic ring [48]. The most characteristic bonds of PVP are located at 1435–1450 cm^−1^ and are attributed to the C-N bond of the aromatic ring [30]. The identification of these bands confirms that the obtained nanofibers were from PVP. In the RPET/PVP spectrum, a band at 3442 cm^−1^ was observed, which is attributed to the characteristic vibration of the O-H bonds of RPET and PVP. The vibration peaks of the C=O bond are at 1720 cm^−1^ and 1648 cm^−1^, which correspond to the double bonds in RPET and PVP, respectively. At 1435 cm^−1^, the characteristic vibrations of the C-N of PVP were present. At 2960 cm^−1^, 1100 cm^−1^, and 730 cm^−1^, the characteristic vibrations of the C-H, C-O-C, and C-H bonds of RPET were also present. Finally, the FTIR spectrum of RPET/PVP/ST showed the same bands as the RPET/PVP spectrum; the molecular vibration of the ST molecule was not appreciable. However, the molecular vibrations of PVP and RPET were observed, confirming the formation of a nanocomposite.

### 3.4. TGA/DSC

**Figure 5:**
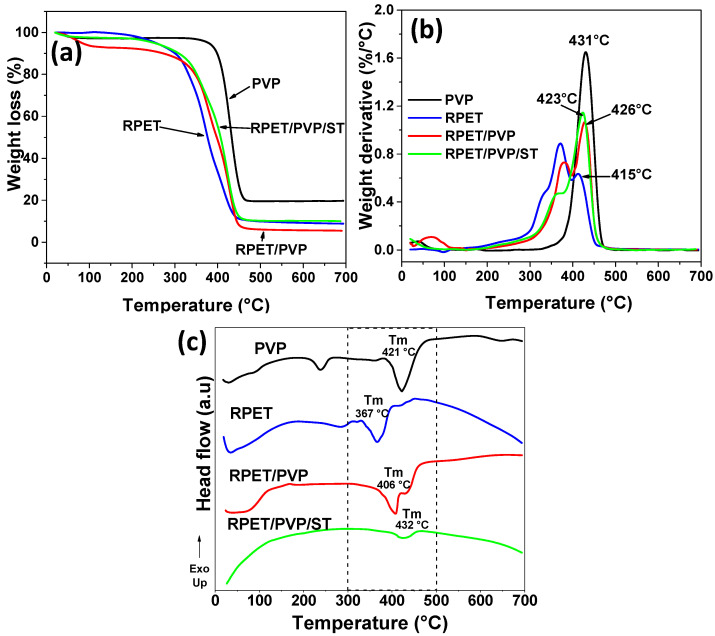
Thermal analysis. (**a**) TGA, (**b**) DTGA, and (**c**) DSC of the electrospun nanofibers.

Figure 5 shows the thermal study results of RPET, PVP, RPET/PVP nanofibers, and RPET/PVP/ST nanofibers. Figure 5a shows the weight loss versus temperature (TGA), Figure 5b shows the derivative of weight versus temperature, and Figure 5c shows the DSC.

The most significant RPET weight losses (Figure 5a), which occurred at 383 °C and 470 °C, are attributed to the noncrosslinked oligomers and to the degradation of the ester group chain (-CO-C) and the unsaturated chain (-C=C-) [49]. The PVP nanofibers showed a weight loss between 20 °C and 105 °C due to the evaporation of physically bound water [50]. At 400–480 °C, further weight loss occurred, which is attributed to the structural decomposition of PVP [51,52]. It can be observed that the thermal behavior of the composite nanofibers is similar to that of PVP and RPET. Specifically, the weight loss of PVP and RPET was 80.3 and 91.5%, respectively; the weight loss of RPET/PVP nanofibers was 94.8%; and the RPET/PVP/ST nanofibers showed a weight loss of 90.1%, which indicates that, under the same conditions, these nanofibers are more thermally stable. This can be attributed to the crosslinking that occurs with the ST monomers [16]. Figure 5b shows peaks where greater weight losses occurred in RPET and PVP, as confirmed by TGA. For the composite nanofibers, the first loss was observed at 383 °C, which is attributed to the nonreticulated oligomers of the RPET. The intensity decreased in the ST nanofibers because it functioned as a crosslinking generator, thereby decreasing the number of unreacted oligomers [10]. The second loss was observed at approximately 423–426 °C, which is attributed to the degradation of PVP. Finally, in the DSC (Figure 5c), the PVP nanofibers showed the first endothermic peak at 236 °C, which is associated with the melting temperature of the crystal line phase inside the polymer [53] and a definitive melting temperature (Tm) at 421 °C, and the Tm of the RPET was 367 °C. Furthermore, the RPET/PVP nanofibers presented a Tm of 406 °C; hence, the melting temperature of the RPET was increased. Finally, the RPET/PVP/ST nanofibers showed a Tm of 432 °C; hence, the thermal stability was increased, which corroborated that the composite nanofibers were more resistant to temperature.

### 3.5. Nanoindentation

The results of the reduced modulus of elasticity and the hardness of the PVP, RPET/PVP, and RPET/PVP/ST samples that were obtained by nanoindentation are presented in Table 1. For the PVP nanofibers, a modulus of elasticity of 0.6 ± 0.05 GPa and a hardness of 0.03 ± 0.005 GPa were obtained; these values are similar to the values that were reported in the literature [54,55,56]. For the RPET/PVP nanofibers, the elastic modulus and hardness increased to 5.8 ± 1.02 GPa and 0.29 ± 0.05 GPa, respectively. Finally, the RPET/PVP/ST nanofibers achieved the best results, with a reduced elastic modulus of 17.73 ± 2.13 GPa and a hardness of 0.62 ± 0.1 GPa. These improvements in the mechanical properties of the composite nanofibers were due to the aforementioned rheological properties, as demonstrated by FTIR and TGA; RPET, PVP, and ST, which were chemically bonded, formed a cross-linked nanocomposite with better mechanical properties [21].

### 3.6. Reaction Mechanism of RPET/PVP/ST

**Figure 6:**
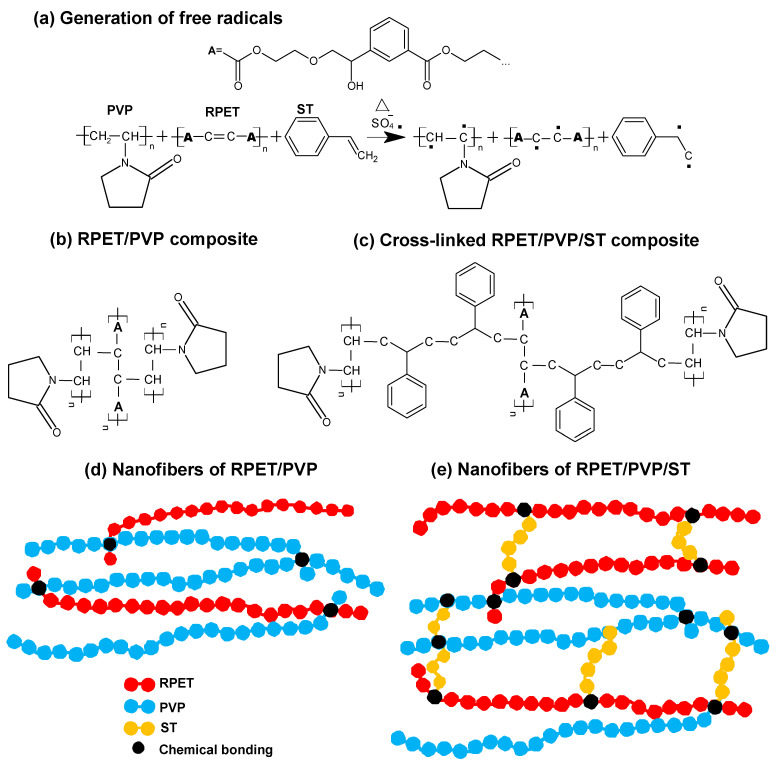
Mechanism of the reaction of nanofibers: (**a**) Generation of free radicals, (**b**,**c**) semideveloped chemical interactions, and (**d**,**e**) schematic representations of composite nanofibers.

KPS is an oxidizing agent that is widely used as a reaction initiator because when it is subjected to a high temperature (catalyst), it transforms into highly reactive sulfate (SO_4_) radicals, which break the bonds in other molecules, especially unsaturated bonds (C=C), leaving these molecules with free radicals and causing a chain polymerization reaction [57,58]. In Figure 6, two reaction mechanisms are proposed among RPET, PVP, and ST based on the rheological analysis, FTIR, TGA/DSC, and nanoindentation results. Figure 6a shows the free radicals that are generated in the macromolecules by the broken lower energy bonds of PVP (C-H) [21] and the unsaturation of RPET and ST (C=C) [59]. These molecules are very reactive and seek to stabilize by covalent bonding until the formation of a composite material (see Figure 6b,c). RPET and PVP can be joined linearly, but the RPET/PVP/ST nanofibers can be crosslinked in all directions to form a polymer network (see Figure 6d,e).

## 4. Conclusions

This study demonstrated the production of a new type of nanofiber from recycled PET and the preparation of RPET/PVP nanofibers without defects and with an average diameter of 300–400 nm with enhanced mechanical properties that were more than 10 times better than those of pure PVP. Finally, crosslinked composite nanofibers of RPET/PVP/ST were obtained. Addition of RPET and ST improved the thermal properties and successfully increased the modulus of elasticity and hardness by up to 29 times compared with those of neat PVP nanofibers; these values are better than those of other polymers that have been reported in the literature. The use of recycled PET to create a new material with better mechanical and thermal performance has great potential for a wide range of applications. In addition, a new reaction mechanism among RPET, PVP, and ST was proposed.

## Figures and Tables

**Table 1:** Reduced moduli and hardnesses of PVP, RPET/PVP, and RPET/PVP/ST nanofibers.

	PVP	RPET/PVP	RPET/PVP/ST
Reduced Elastic Modulus (GPa)	0.60	5.80	17.73
Standard Deviation	0.05	1.02	2.13
Hardness (GPa)	0.030	0.290	0.620
Standard Deviation	0.005	0.05	0.1

## Data Availability

Not applicable.
